# The Role of SCARA5 as a Potential Biomarker in Squamous Cell Carcinoma of the Lung

**DOI:** 10.3390/ijms25137355

**Published:** 2024-07-04

**Authors:** Fidelis Andrea Flockerzi, Johannes Hohneck, Frank Langer, Wolfgang Tränkenschuh, Phillip Rolf Stahl

**Affiliations:** 1 Department of Pathology, Saarland University Medical Center, 66421 Homburg, Germany; 2 Department of Thoracic and Cardiovascular Surgery, Saarland University Medical Center, 66421 Homburg, Germany; 3 Department of Pathology, Medical School Berlin, 14197 Berlin, Germany

**Keywords:** SCARA5, squamous cell carcinoma, lung cancer

## Abstract

Lung cancer is the leading cause of cancer-related deaths in the western world. Squamous cell carcinoma is one of the most common histological subtypes of this malignancy. For squamous cell carcinoma of the lung (LSCC), prognostic and predictive markers still are largely missing. In a previous study, we were able to show that the expression of THSD7A shows an association with unfavorable prognostic parameters in prostate cancer. There is also a link to a high expression of FAK. There is incidence that SCARA5 might be the downstream gene of THSD7A. Furthermore, there is evidence that SCARA5 interacts with FAK. We were interested in the role of SCARA5 as a potential biomarker in LSCC. Furthermore, we wanted to know whether SCARA5 expression is linked to THSD7A positivity and to the expression level of FAK. For this reason, we analyzed 101 LSCC tumors by immunohistochemistry. Tissue microarrays were utilized. No significant association was found between SCARA5 expression and overall survival or clinicopathological parameters. There was also no significant association between THSD7A positivity and SCARA5 expression level. Moreover, no significant association was found between FAK expression level and SCARA5 expression level. SCARA5 seems not to play a major role as a biomarker in squamous cell carcinoma of the lung.

## 1. Introduction

Lung cancer is a very frequent type of human cancer, and in the western world, it is the leading cause of cancer-related deaths [1]. Several histological subtypes of lung cancer are known, with squamous cell carcinoma representing one of the most common. In the past, squamous cell carcinoma of the lung occurred much more frequently in men, but lately, the number of women suffering from LSCC has noticeably increased. In the meantime, several predictive markers have been established for adenocarcinomas of the lung. For this reason, a certain number of patients are eligible to receive targeted therapy for this cancer subtype. For squamous cell carcinoma of the lung (LSCC), prognostic and predictive markers are largely missing. There might be exceptions like PD-L1, rare mutations in Epidermal Growth Factor Receptor (EGFR), and rearrangements in Anaplastic Lymphoma Kinase (ALK) [2,3,4]. Nonetheless, the prognosis of LSCC remains poor. In particular, to avoid the well-known side effects of standard chemotherapy and to improve prognosis, the establishment of novel predictive markers allowing for targeted therapy is urgently needed. This might also be important for subgroups of patients (e.g., gender).

Recently, we showed that Thrombospondin Type-1 Domain containing 7A (THSD7A) is associated with poor overall survival in female patients with LSCC. Furthermore, THSD7A positivity was linked to high expression of Focal Adhesion Kinase (FAK) and FGFR1 wildtype in this subgroup [5].

THSD7A is involved in the pathogenesis of membranous nephropathy (MN) [6,7,8]. Apart from that, THSD7A might also play a role in cancer. Several groups reported on the potential role of THSD7A as a prognostic marker in different tumor types [9,10,11,12,13]. Furthermore, there is a possible relationship between THSD7A expression in different tumor types and MN [6,14,15,16,17,18,19]. Furthermore, THSD7A possibly activates Focal Adhesion Kinase (FAK) [20,21,22]. FAK is a protein tyrosine kinase regulating cellular adhesion, survival and proliferation in different types of cells. FAK shows overexpression in various tumor types and probably plays a role in tumor progression and metastasis [23,24,25,26]. For this reason, FAK is currently quite an established tumor marker and might serve as a cancer target in several tumors [27,28,29,30,31]. Recently, we reported on the association between THSD7A positivity and high expression of FAK in prostate cancer [32]. This finding underlines the possible involvement of THSD7A in FAK-dependent signaling pathways, potentially describing an independent mode of tumor development. 

In a previous study, Jumai et al. came to the conclusion that in esophageal squamous cell carcinoma (ESCC), scavenger receptor class A member 5 (SCARA5) might be a downstream gene of THSD7A [33]. Until now, not much data are available dealing with SCARA5. Scavenger receptors are described as a superfamily of membrane-bound receptors. Scavenger receptor class A has five known members. They are type II transmembrane proteins, enabled to form homotrimers on the cell surface and to recognize various ligands. They seem to be involved in different biological pathways, particularly influencing host defense and ferritin homeostasis [34]. 

Moreover, there is evidence that SCARA5 is involved in tumor development and progression. Most researchers describe SCARA5 as a tumor suppressor by inhibiting PI3K/AKT and ERK/MAPK pathways, two main pathways leading to cellular growth and proliferation. Investigations with these results can be found for several tumor entities, for example renal cell carcinoma, hepatocellular carcinoma, colorectal cancer, lung cancer, breast cancer, oral squamous cell carcinoma, gastric cancer and osteosarcoma [35,36,37,38,39,40,41,42,43,44,45,46].

In addition, the results of several groups indicate that SCARA5 might directly interact with FAK. For example, Wen et al. could show that the overexpression of SCARA5 inhibits tumor invasion and proliferation in osteosarcoma. They also state that the phosphorylation of FAK and therefore its activation are inhibited by SCARA5 overexpression in this malignancy [45].

A similar observation was made by Yan et al. They report that the upregulation of SCARA5 in human glioma cell lines significantly reduces phosphorylation of FAK [47].

Huang et al. indicate that SCARA5 might inhibit the activation of the FAK-Src-Cas pathway in hepatocellular carcinoma and therefore can be discussed as a tumor suppressor in this entity [41].

Lee et al. state that SCARA5 plays a role in the adipocyte lineage commitment via involvement of the FAK-ERK pathway [48].

In a recent study, we were able to demonstrate that high SCARA5 expression is significantly associated with an advanced tumor stage, with positive nodal status and with a high Gleason Score in prostate cancer. No significant association was found between the expression levels of SCARA5 and FAK. We also did not find a significant association between the expression level of SCARA5 and THSD7A positivity when utilizing our defined score. However, prostate cancers with strong SCARA5 positivity were associated with THSD7A positivity [49]. 

Against that background, there is evidence that SCARA5 plays a potential role in tumor development and might be part of a potential THSD7A-driven mode of tumorigenesis. Furthermore, SCARA5 might be involved in FAK-dependent signaling pathways.

To our knowledge, no data exist on the association between SCARA5 expression and clinicopathological parameters in LSCC so far.

The aim of this study was to evaluate the association between the expression of SCARA5 and common clinicopathological parameters in squamous cell carcinoma of the lung. Further, we were interested in the association of SCARA5 expression with THSD7A positivity and with the expression levels of FAK in its unphosphorylated form, respectively.

Therefore, a previously described cohort of 101 LSCC tumors [50] was examined by immunohistochemistry (IHC) using tissue microarrays (TMAs).

This previous study dealt with Fibroblast growth factor receptor 1 (FGFR1). We demonstrated that amplification of FGFR1 is a frequent event in LSCC. Amplification of FGFR1 showed a significant association with late tumor stage. In female patients, FGFR1 amplification was associated with better overall survival.

## 2. Results

**Figure 1:**
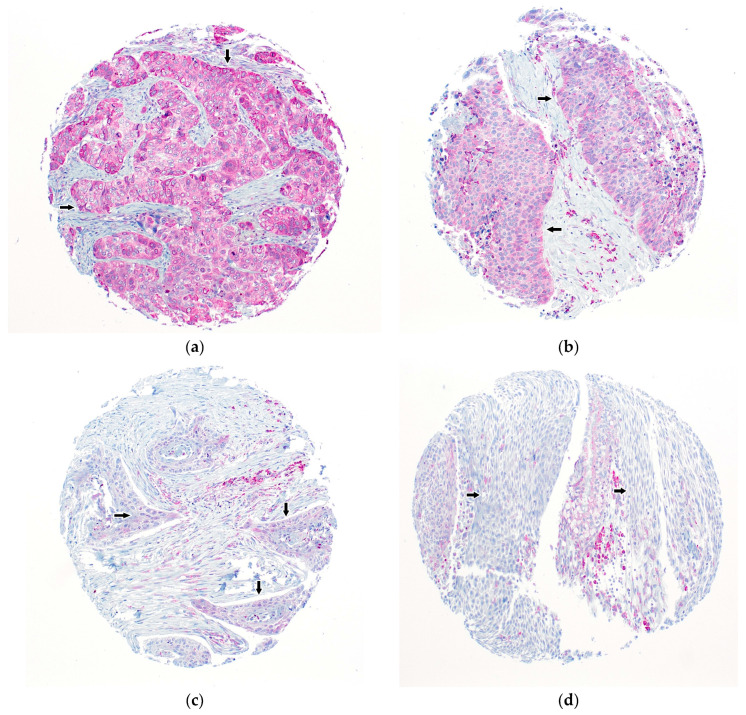
SCARA5, cytoplasmic staining pattern: (**a**) 3+ staining, (**b**) 2+ staining, (**c**) 1+ staining, (**d**) negative tumor tissue, magnification 100×. Arrows mark the tumor cells.

Seventy-four (73.3%) tumors were analyzable for SCARA5-IHC. Twenty-seven (26.7%) tumors were not analyzable due to a total lack of tissue or due to a lack of unequivocal tumor tissue. Thirty-one (41.9%) tumors showed high SCARA5 expression and forty-three (58.1%) tumors showed low SCARA5 expression. SCARA5 showed a cytoplasmic staining pattern. Non-tumor tissue was mainly negative for SCARA5 and showed at best a very weak staining intensity. Representative images are shown in Figure 1.

Seventeen female patients were analyzable for SCARA5-IHC; seven (41.2%) showed high SCARA5 expression. Fifty-seven male patients were analyzable for SCARA5-IHC; twenty-four (42.1%) of them showed high SCARA5 expression.

**Figure 2:**
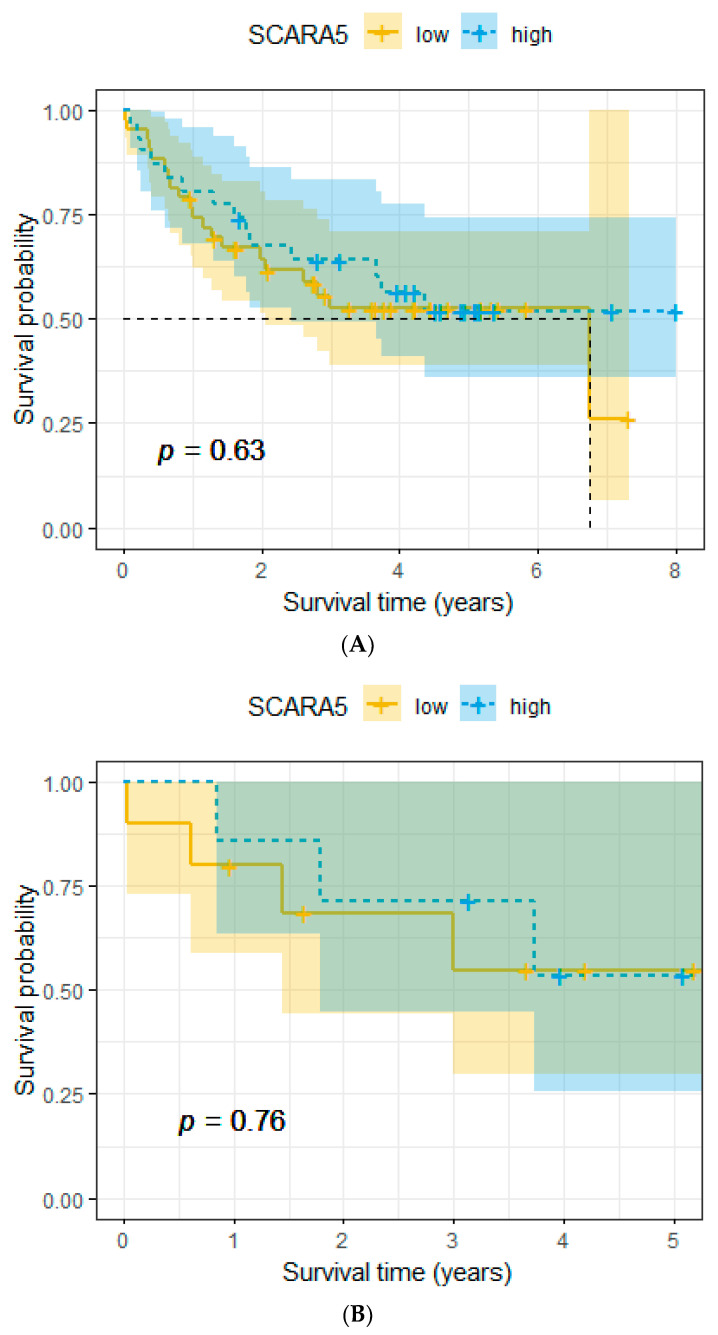

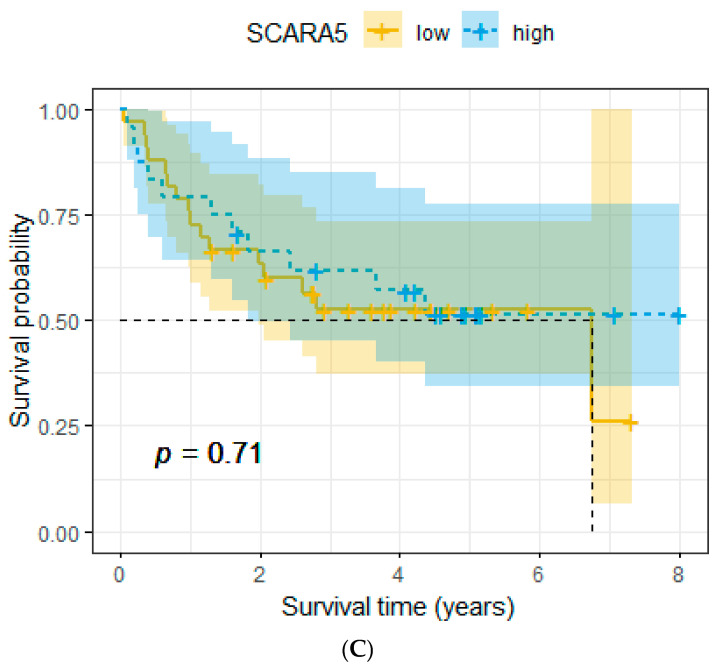
(**A**) Association between expression levels of SCARA5 and overall survival (whole cohort). (**B**) Association between expression levels of SCARA5 and overall survival (female subgroup). (**C**) Association between expression levels of SCARA5 and overall survival (male subgroup).

We did not find a significant association with overall survival. This applies for the whole cohort as well as for the subgroups of female patients and male patients (*p* = 0.63, *p* = 0.76, and *p* = 0.71, respectively, Figure 2A–C). 

In addition, we could not find a significant association between SCARA5 expression levels and clinicopathological parameters. The results are shown in detail in Table 1.

### 2.1. Association between Expression Levels of SCARA5 and THSD7A Positivity

To demonstrate a potential relationship between SCARA5 and THSD7A in LSCC, we compared the expression levels of SCARA5 with previously collected data on THSD7A. Seventy-four (73.3%) tumors were analyzable for THSD7A-IHC as well as for SCARA5-IHC. We did not find a significant association between expression levels of SCARA5 and THSD7A positivity (*p* = 0.7). Table 2 shows the results.

### 2.2. Association between Expression Levels of SCARA5 Expression and FAK Expression

To demonstrate a potential relationship between expression levels of SCARA5 and FAK in LSCC, we compared SCARA5 expression with previously collected data on FAK. Seventy-four (73.3%) tumors were analyzable for FAK-IHC as well as for SCARA5-IHC. We did not find a significant association between expression levels of SCARA5 and FAK expression levels (*p* = 0.5). Table 3 shows the results.

### 2.3. Association between Expression Levels of SCARA5 and FGFR1 Amplification Status

To demonstrate a potential relationship between expression levels of SCARA5 and FGFR1 amplification in LSCC, we compared SCARA5 with our previously collected data on FGFR1. Seventy-four (73.3%) tumors were analyzable for FGFR1 as well as for SCARA5-IHC. We did not find a significant association between expression levels of SCARA5 and FGFR1 amplification (*p* = 0.4). Table 4 shows the results.

## 3. Discussion

Squamous cell carcinoma is a frequent histological subtype of lung cancer. For this tumor entity, there is still quite a lack of prognostic and predictive markers, especially if you look at the developments in adenocarcinoma of the lung. With respect to the poor prognosis of LSCC, there is an urgent need for novel predictive (molecular) markers and targets.

THSD7A shows a significant association with unfavorable prognostic parameters in prostate cancer. THSD7A positivity is also associated with early PSA recurrence and with high FAK expression in this malignancy [9,32]. 

Recently, it was reported that in esophageal squamous cell carcinoma, scavenger receptor class A member 5 might be the downstream gene of THSD7A. Furthermore, the authors stated that SCARA5 promotes the migration and proliferation of cancer cells and pointed out that expression levels of SCARA5 are different between ESCC and normal esophageal tissue. However, no significant association between SCARA5 expression and clinicopathological parameters or prognosis could be demonstrated [33]. Interestingly, SCARA5 might also directly interact with FAK. There is some evidence that SCARA5 overexpression inhibits the phosphorylation and thus the activation of FAK in several tumor types. Data regarding this assumption are, for example, available for osteosarcoma and hepatocellular carcinoma [45,47]. Additionally, Lee et al. describe an essential role of SCARA5 in the adipocyte lineage commitment via involvement of the FAK-ERK signaling pathway [48]. This also indicates an interaction between SCARA5 and FAK.

In view of the above, we were interested in the role of SCARA5 as a potential biomarker in LSCC. Furthermore, we were interested in a potential connection between SCARA5 expression and the expression status of THSD7A and FAK, respectively.

In our study, no significant association was found between SCARA5 expression and clinicopathological parameters as well as overall survival. This applied for the whole population as well as for the female and male subgroups. We also could not find a significant association between expression levels of SCARA5 and THSD7A positivity. Furthermore, we did not find a significant association between expression levels of SCARA5 and FAK expression. 

Most previous studies on SCARA5 describe this biomarker as a tumor suppressor gene. Many reports on the downregulation of SCARA5 in different tumor types can be found, for example, breast cancer, oral squamous cell carcinoma, colorectal cancer, renal cell carcinoma, and gastric cancer [35,36,37,38,39,40,42,43,44,46,51,52,53].

Liu J et al. report on a downregulation of SCARA5 mRNA levels in colorectal cancer tissues in comparison with normal tissue, and they state that low expression of SCARA5 is linked to poor prognosis. These results were validated in clinical specimen using IHC [51]. Khamas et al. report on down-expression of SCARA5 in tumor tissue and in colorectal cancer cell lines compared to non-tumor tissue [43]. According to Liu H et al., low-level expression of SCARA5 is correlated with tumor size, tumor stage and venous metastasis in hepatocellular carcinoma. Furthermore, they report on a significantly better survival in the group with high expression of SCARA5 [52]. A significant downregulation of SCARA5 in breast cancer tissues and cells as well as a correlation with tumor size, histological grade and lymph node metastasis was reported by You et al. They also state that overexpression of SCARA5 suppresses invasion and cell proliferation [44]. Ulker et al. demonstrated decreased SCARA5 expression in breast cancer tissue compared to non-tumor tissue. They could also detect an association between SCARA5 expression with histological grade [37]. For gastric cancer, similar results were reported. Zhang et al. could show an inverse correlation between SCARA5 protein levels and aggressive clinicopathological characteristics and with poor prognosis in this tumor entity. They also state that the overexpression of SCARA5 suppresses the migration, growth, and invasion of gastric cancer cell lines in vitro and that upregulation of SCARA5 inhibits metastasis and tumor growth in a xenograft model [35]. Decreased SCARA5 expression in oral squamous cell carcinoma in comparison to normal oral mucosa was reported by Liu Y et al. They also state that SCARA5 downregulation is associated with invasion and cell proliferation [38]. Ni et al. report on lower SCARA5 mRNA expression in melanoma than in adjacent normal skin. In this study, decreased SCARA5 expression was correlated with tumor stage and nodal status as well as with recurrence and metastasis. It was shown that tumors with high expression of SCARA5 showed a significantly better overall survival in comparison to tumors with low expression of SCARA5 [53].

Our results more or less differ from previously collected data. We cannot confirm SCARA5 as a tumor suppressor gene in LSCC. We did not find a significant association between SCARA5 expression and either clinicopathological parameters or overall survival. 

In one study, SCARA5 was described as a downstream gene of THSD7A in esophageal squamous cell carcinoma. It was also stated that SCARA5 expression is significantly higher in tumor tissue in comparison to normal adjacent tissue [33]. We could not demonstrate a connection between expression levels of SCARA5 and THSD7A positivity in LSCC. 

Some authors report on a potential interaction between SCARA5 and FAK. However, we did not find a connection between expression levels of SCARA5 and FAK expression levels.

Furthermore, we did not find a significant association between expression levels of SCARA5 and FGFR1 amplification.

A weakness of our study is that we only analyzed a quite small cohort. Furthermore, we conducted this analysis as a pure IHC study, also for reasons of feasibility. Certainly, no conclusions can be drawn regarding functional interactions between the evaluated markers and parameters.

To our knowledge, we are the first to report on the association of SCARA5 with clinicopathological parameters and overall survival in LSCC. 

In addition, we were able to compare SCARA5 expression with THSD7A positivity, FAK expression level and FGFR1 gene amplification, respectively.

## 4. Materials and Methods

### 4.1. Materials

A total of 101 primary LSCC tumors were acquired in a tissue microarray format. This cohort was described previously. All patients underwent surgical treatment at the Department of Thoracic and Cardiovascular Surgery of the University Hospital of Saarland, Germany. Surgery took place between 2006 and 2013. Table 1 shows detailed clinicopathological parameters.

### 4.2. Tissue Microarrays

Construction of the TMA was performed according to the manufacturers’ directions (Manual Tissue Arrayer, AlphaMetrix Biotech, Rödermark, Germany). A Manual Tissue Arrayer was used. In correlation with corresponding H&E-stained tissue slides, paraffin-embedded tumor tissue blocks were selected. With hollow needles (diameter of 1.0 mm), tissue cylinders were punched out of the selected tumor tissue blocks. These tumor tissue blocks served as “donor blocks”. Before each withdrawal of tissue from the donor block, a hole was punched out of an empty paraffin block. This block served as the “recipient block”. The tissue cylinder from the donor block was then brought into the empty hole of the “recipient” paraffin block. This technology allows for simultaneous analysis of all included tissue samples. 

Then, 4 µm sections of the TMA blocks were cut. These were transferred to adhesion slides (Matsunami TOMO, Osaka, Japan) and were used for IHC. 

### 4.3. Immunohistochemistry

To transfer the fresh-cut sections to adhesive slides, a water bath (46 °C) was used. Then, the sections were dried overnight at 37 °C. A primary antibody specific for SCARA5 (dilution: 1:150; rabbit polyclonal antibody, abcam; cat# ab118894) was used. To perform staining, Benchmark Ultra (Ventana Medical Systems/Roche, Basel, Switzerland) was utilized.

The bound antibody was visualized using ultraView Universal Alkaline Phosphatase Red Detection (Roche, Basel, Switzerland) according to the manufacturers’ directions. For heat-induced antigen retrieval, CC1 buffer (Ventana/Roche, Basel, Switzerland) was used at 95 °C for 64 min.

The expression of SCARA5 was evaluated by estimating the percentage of positive tumor cells. The staining intensity was recorded semiquantitatively for each tissue sample (0, 1+, 2+, 3+). Tumors that showed no staining (0) were considered negative. To our knowledge, no validated scoring system for SCARA5 expression exists. To achieve a clear-cut evaluation, SCARA5 expression was dichotomized as being low-level (tumors with 0 staining, 1+ staining ≤70% and 2+ staining in ≤30% of tumor cells) and high-level (tumors with 1+ staining in >70%, 2+ staining in >30% of tumor cells and 3+ staining in any tumor cell).

### 4.4. Statistics

Statistical analysis was performed using R (R Corporation 2021, R Foundation for Statistical Computing). Pearson’s Chi-squared test (with Yate’s correction for continuity) was used for testing the null hypothesis of independence of two categorical variables. Kaplan–Meier curves and the log-rank test were used to test for differences in survival time between the subgroups.

## 5. Conclusions

To our knowledge, we are the first to investigate the role of SCARA5 as a potential tumor marker in squamous cell carcinoma of the lung. We did not find a significant association between SCARA5 expression and either clinicopathological parameters or overall survival. We also did not find a significant association between SCARA5 expression and THSD7A positivity and FAK expression levels, respectively. In contrast to other tumor entities, SCARA5 appears to not show characteristics of a tumor suppressor gene in squamous cell carcinoma of the lung. 

## Figures and Tables

**Table 1:** Association between expression levels of SCARA5 and clinicopathological parameters.

	SCARA5		*p*-Value
	Low	High	
**Sex**			>0.9
female	10	7	
male	33	24	
**Histology**			0.8
non-keratinizing	28	21	
keratinizing	15	10	
**Tumor status**			0.3
pT1	7	10	
pT2	19	10	
pT3	11	5	
pT4	6	6	
**Nodal status**			0.3
pN0	23	20	
pN+	20	11	
**Grading**			0.1
G1	0	0	
G2	17	19	
G3	26	12	

**Table 2:** Association between expression levels of SCARA5 and THSD7A positivity.

	SCARA5		*p*-Value
	Low	High	
**THSD7A**			0.7
negative	38	29	
positive	5	2	

**Table 3:** Association between expression levels of SCARA5 and FAK expression.

	SCARA5		*p*-Value
	Low	High	
**FAK**			0.5
low	36	24	
high	7	7	

**Table 4:** Association between expression levels of SCARA5 and FGFR1 amplification.

	SCARA5		*p*-Value
	Low	High	
**FGFR1**			0.4
no amplification	34	22	
amplified	9	9	

## Data Availability

Analyzed and used datasets are available from the corresponding author on reasonable request.
